# Patients’ perception and needs of spiritual care: A qualitative study in the context of prolonged hospitalizations

**DOI:** 10.1371/journal.pone.0347552

**Published:** 2026-04-24

**Authors:** Rahime Aydin Er, Gamze Çetiner, Yüksel Can Öz

**Affiliations:** 1 Department of Psychiatric Nursing, Kocaeli University Faculty of Health Sciences, Kocaeli, Türkiye; 2 Department of History of Medicine and Ethics, Kocaeli University School of Medicine, Kocaeli, Türkiye; 3 Orthopedics and Traumatology Clinic, Kocaeli City Hospital, Kocaeli, Türkiye; Queensland University of Technology - QUT: Queensland University of Technology, AUSTRALIA

## Abstract

Spiritual care is recognized as a core component of holistic nursing, particularly for patients experiencing prolonged hospitalization. Extended hospital stays may intensify spiritual needs by disrupting daily routines, limiting engagement in religious practices, and increasing existential distress. However, empirical evidence on patients’ spiritual care needs and their perceptions of nursing practices remains limited. This study aimed to explore patients’ spiritual care needs during prolonged hospitalization and to examine their perceptions of spiritual care provided by nurses. A qualitative study with a descriptive phenomenological design was conducted. Semi-structured, in-depth interviews were carried out with 12 patients hospitalized in internal medicine clinics for more than 14 days. Data were analysed using an inductive thematic analysis approach. Participants described spirituality as encompassing both religious practices and broader dimensions such as inner peace, psychological balance, and meaning making. Spiritual needs were frequently reflected in participants’ use of religious rituals, including prayer, ablution, and supplication, reflecting a desire for closeness to God. In addition, nurse–patient communication, empathetic attitudes, psychosocial support, and assistance with personal hygiene were identified as integral components of spiritual care. Environmental and institutional factors, including shared rooms, limited access to prayer spaces, and inadequate hygiene conditions, emerged as significant barriers to meeting spiritual needs. The findings demonstrate that spiritual care in prolonged hospitalization is a multidimensional process encompassing relational, physical, and environmental dimensions of care. Addressing spiritual needs requires both compassionate communication and supportive clinical environments that enable patients to sustain their spiritual practices. Integrating spiritual care into routine nursing practice may enhance patient experience and strengthen the holistic nature of care.

## Introduction

Spirituality is widely recognized in nursing scholarship as an individual’s enduring search for meaning, purpose, and connection with self, others, the environment, and, for many, a transcendent or divine presence [1]. As such, it constitutes a fundamental dimension of human experience and an essential component of holistic nursing care, which seeks to integrate physical, psychosocial, and spiritual aspects of health [2,3]. Importantly, spirituality is not a uniform construct; rather, it is shaped by cultural, religious, and socio-contextual factors, leading to diverse expressions and care needs across populations and settings [4].

Within holistic care frameworks, attention to spiritual needs has been associated with improved psychological well-being, enhanced coping with illness, and higher quality of life [5–8]. Conversely, the neglect of spiritual concerns has been linked to decreased patient satisfaction and suboptimal recovery trajectories [9]. These findings underscore that spiritual care is not an optional or peripheral aspect of nursing practice, but a core element of patient-centered and whole-person care.

Conceptually, spiritual needs have been described as multidimensional. Spiritual needs have been conceptualized as encompassing situational (related to care context and relationships), biographical (meaning making and life narrative), moral (values, dignity, and ethical concerns), and religious (belief systems and practices) dimensions [10]. This framework provides a useful basis for interpreting how patients experience and articulate spirituality within clinical environments, particularly in contexts where religious and cultural elements are closely intertwined.

Hospitalization, especially when prolonged, represents a critical context in which spiritual needs may become more visible and intensified. Extended hospital stays disrupt daily routines, limit engagement in meaningful activities, restrict access to social support, and may hinder the performance of religious or spiritual practices [11–13]. Such disruptions can give rise to feelings of uncertainty, loss of control, loneliness, and existential distress. At the same time, spiritually supportive interactions and environments may help patients reconstruct meaning, sustain hope, and maintain a sense of connectedness [13–15].

Nurses, as the primary providers of continuous bedside care, are uniquely positioned to recognize and respond to patients’ spiritual needs. Through therapeutic communication, empathetic presence, and sensitivity to individual beliefs and values, nurses can integrate spiritual care into everyday clinical practice [16,17]. Despite its recognized importance and its inclusion in professional and ethical standards [18], spiritual care continues to be inconsistently implemented, often overshadowed by biomedical priorities and constrained by time limitations, insufficient training, and organizational barriers [19–21].

The sociocultural context in which care is delivered plays a crucial role in shaping how spirituality is understood and enacted. As a secular republic with a largely Muslim cultural background, Türkiye represents a context in which multiple understandings of spirituality coexist. It reflects a context where secular and religious influences coexist and shape how spirituality is understood.In such settings, spirituality may reflect both religious practices and culturally embedded forms of meaning-making that are influenced by Islamic traditions, even when not explicitly articulated as religious [22]. However, empirical research examining how patients in Türkiye conceptualize spirituality, express spiritual needs, and evaluate spiritual care within hospital settings remains limited [23,24].

This gap is especially pronounced in the context of prolonged hospitalization, where patients’ spiritual vulnerability may increase and their expectations of care extend beyond physical treatment to include relational, emotional, and spiritual dimensions. Understanding lived experiences of patients in this context is essential for developing culturally sensitive and clinically meaningful models of spiritual care. Accordingly, the present study aimed to explore the spiritual care needs of patients experiencing prolonged hospitalization and to examine their perceptions of the spiritual care provided by nurses.

## Methods

### Study design

Given that spiritual care is grounded in subjective meaning-making processes and lived experience rather than objectively measurable variables, this study adopted a qualitative phenomenological design to explore patients’ perceptions of spiritual care during prolonged hospitalization. A descriptive phenomenological approach was employed to capture the essence of participants’ experiences and to remain close to their accounts without imposing prior interpretations. This approach is well suited to examining how individuals interpret and assign meaning to complex phenomena such as spirituality within a specific clinical context, while allowing both shared patterns and individual variations to emerge [25]. The study was reported in accordance with the Consolidated Criteria for Reporting Qualitative Research (COREQ) checklist to ensure transparency and completeness [26] (S1 Appendix: COREQ checklist).

### Study setting and sample

This study was conducted in two public hospitals located in a densely populated and industrialized city in western Türkiye, characterized by high population mobility. Internal medicine clinics were selected as the study setting because they commonly accommodate patients with prolonged hospitalizations—defined as stays longer than 14 days [27]—during which disruptions in daily routines, social roles, and access to personal spiritual practices tend to intensify, making spiritual needs more salient.

Participants included patients aged 18 years or older who had been hospitalized for at least 15 days, were not bedridden, and voluntarily agreed to participate. To facilitate in-depth exploration of experiences, individuals with visual or hearing impairments that could hinder communication were excluded.

Although there is no strict rule for sample size in qualitative research, adequacy is determined by the depth and richness of the data rather than numerical thresholds. In this study, recruitment continued until thematic sufficiency (data saturation) was achieved—that is, when additional interviews did not yield substantially new insights and existing themes were well developed [25]. This point was reached after the 12th participant, at which stage data collection was concluded.

### Data collection tools

Data were collected using a semi-structured interview guide developed by the researchers based on a review of the relevant literature and the study objectives. The guide was reviewed by researchers experienced in qualitative nursing research to ensure clarity, cultural sensitivity, and alignment with the study aims.

The interview guide consisted of two parts. The first part included questions on participants’ demographic (gender, age, marital status, number of children, occupation, employment status, and duration) and clinical (diagnosis, disease duration, number of hospitalizations, and length of current stay) characteristics. The second part comprised open-ended questions exploring participants’ perceptions of spirituality and spiritual care, their spiritual care needs, and their experiences of spiritual care practices in the hospital setting (S2 Appendix: Semi-structured interview protocol).

The semi-structured format provided a flexible yet focused framework, enabling in-depth exploration of participants’ experiences while ensuring consistency across interviews through predefined guiding questions [25].

### Data collection

After obtaining institutional permissions, the first author (a female psychiatric nurse) informed the head nurses of the clinics about the study and accessed potential participants through them. The researcher visited the clinics three times per week (Monday, Wednesday, and Friday) to identify patients who met the inclusion criteria. Eligible patients were verbally informed about the study and provided with an information sheet. Three eligible patients declined participation without providing reasons. Those who agreed were scheduled for interviews during subsequent visits.

All interviews were conducted by the first author, who has experience in qualitative research. Prior to data collection, rapport was established to facilitate open and trust-based communication. Interviews were carried out in patients’ rooms at times that ensured privacy and did not interfere with clinical care. Only the researcher and the participant were present; if a third person entered, the interview was paused. Before each interview, participants were re-informed about the study, and written informed consent was obtained for both participation and audio recording. Field notes were taken during and after interviews to capture contextual details and nonverbal cues that could enrich data interpretation. To maintain participants’ engagement and minimize fatigue, interviews were planned to last approximately 30 minutes; however, actual durations ranged from 19 to 35 minutes depending on participants’ responses. Data collection was conducted between February and September 2023 and continued until thematic sufficiency (data saturation) was achieved.

### Data analysis

The analysis was based on verbatim interview transcripts and detailed field notes. Qualitative data analysis proceeded concurrently with data collection in an iterative process. Each interview was transcribed verbatim within two weeks of completion. Early transcription facilitated immersion in the data and enabled preliminary coding while subsequent interviews were still ongoing. The transcripts and field notes were read repeatedly to achieve familiarity with the data and to identify meaning units relevant to the research aims [25].

Data were analysed manually using an inductive approach based on the analytic procedures. Meaning units were identified and coded inductively in interview transcripts. These initial codes were compared and iteratively grouped into categories, and relationships among categories were examined to generate overarching themes representing meaningful patterns across the dataset [28]. To enhance analytical rigor and minimize researcher bias, the two researchers (RAE, PhD, professor of medical ethics with experience in qualitative health research; GÇ, MSc, RN) conducted independent coding and initial theme development [29]. The independently generated codes and preliminary themes were then compared. In cases where alternative interpretations emerged, third researcher (YCÖ, PhD, psychiatric nurse with experience in qualitative research) participated in joint analytic discussions. All discrepancies were reviewed collaboratively, and consensus was reached through discussion. This process constituted analyst triangulation, strengthening the credibility and dependability of the findings by incorporating multiple analytic perspectives [30]. In addition, regular analytic meetings were held among the research team to critically review coding decisions and emerging themes, thereby enhancing methodological rigor and analytic transparency [31]. Final themes are presented with verbatim participant quotations to preserve the authenticity of participants’ accounts and to enable readers to evaluate the relationship between the data and the interpretations. Quantitative data were analysed using descriptive statistics (frequency and percentage).

### Reflexivity

The research team consisted of researchers with backgrounds in nursing and medical ethics, which shaped the approach to data collection and interpretation. Throughout the research process, reflexivity was maintained by critically reflecting on researchers’ preconceptions, professional roles, and potential influence on participants and the analytic process.

During data collection, particular attention was paid to establishing a neutral and non-judgmental interview environment to minimize potential power imbalances. The researcher conducting the interviews adopted a reflective and empathetic stance to facilitate open communication. Field notes and reflexive notes were used to document contextual observations, emerging impressions, and potential biases. These reflections were continuously considered during data analysis to enhance interpretive awareness and to reduce the influence of researchers’ assumptions on the findings [32].

### Trustworthiness

To ensure the trustworthiness of the study, several strategies were employed in line with established qualitative research criteria, including credibility, dependability, confirmability, and transferability [31]. Credibility was enhanced through prolonged engagement with the data, iterative analysis, and the use of verbatim quotations to support the findings. Analyst triangulation was achieved through independent coding by two researchers and subsequent consensus discussions. Dependability was supported by maintaining a detailed audit trail documenting coding decisions, theme development, and analytic processes. Confirmability was strengthened through reflexive practices and the use of field notes to ensure that findings were grounded in the data rather than researchers’ assumptions. Transferability was addressed by providing detailed descriptions of the study setting, participant characteristics, and data collection procedures, enabling readers to assess the applicability of the findings to other contexts.

### Ethical considerations

The study was conducted in accordance with the principles of the Declaration of Helsinki. Ethical approval was obtained from the Clinical Research Ethics Committee of Derince Training and Research Hospital, University of Health Sciences (date: 12 January 2023; number: 2023−9).

All participants were informed about the study’s purpose, procedures, voluntary nature, and confidentiality and written informed consent was obtained. Participants were informed of their right to withdraw at any time without consequences. All data were stored on a password-protected computer accessible only to the authors. Anonymity was maintained throughout data analysis and reporting, and direct quotations are presented using anonymized identifiers.

## Results

The demographic and clinical characteristics of the 12 patients are summarized in Table 1. Participants ranged in age from 24 to 90 years (mean = 62). The number of hospitalizations varied between one and four, with more than half of the participants (n = 7, 58.3%) hospitalized for the second time. The duration of the current hospital stay ranged from 15 to 120 days, with a mean of 28 days.

**Table 1:** Demographic and clinical characteristics of the participants.

No	Age	Gender	Marital status	Education	Occupation	Diagnosis	Hospitalizations (no)	Illness duration (days)	Current stay (days)
1	24	Male	Single	Associate degree	Worker	Pneumothorax	3	21	21
2	35	Female	Married	Associate degree	Healthcare personnel	Pneumonia	2	18	18
3	48	Female	Single	Secondary school	Hairdresser	Tongue cancer	3	120	120
4	55	Female	Single	Associate degree	Teacher	Amputation infection	4	32	32
5	60	Male	Single	Primary school	Worker	Infection	1	16	16
6	64	Male	Married	Primary school	Self-employed	Laryngeal cancer	1	22	22
7	69	Male	Married	Secondary school	Self-employed	Renal failure	2	17	17
8	71	Female	Married	Primary school	Housewife	Pulmonary edema	2	365	16
9	71	Male	Married	Primary school	Self-employed	Lung cancer	2	120	30
10	80	Male	Married	Primary school	Worker	Lung cancer	2	365	17
11	82	Male	Married	Literate	Worker	Pneumonia	2	15	15
12	90	Male	Single	Primary school	Civil servant	Chronic obstructive pulmonary disease	2	15	15

Thematic analysis identified nine main themes and thirteen subthemes reflecting how patients perceived and experienced spiritual care during prolonged hospitalization (Table 2). These themes encompass patients’ understandings of spirituality, their spiritual needs, the personal strategies they used to address these needs, and their expectations of nursing care.

**Table 2:** Themes, subthemes, and illustrative codes derived from qualitative analysis.

Theme	Subtheme	Illustrative codes
Perception of spirituality	Inner peace and comfort	Peace, inner calm, happiness, stressless, feeling well
	Faith-based definitions	Faith, closeness to God, prayer, worship
Perception of spiritual care	Therapeutic relationship and attention	Caring, helping, supporting, promoting healing, providing comfort, providing peace
	Effective communication	Smiling, kind communication, sincerity, non-judgement, listening, boosting morale
	Association with faith	Closeness to God, spiritual strengthening, facilitation of worship practices
Spiritual needs during hospitalization	Psychosocial support	Family presence, visits, emotional sharing, motivation, being asked about well-being
	Personal hygiene	Bathing, showering, cleansing, ablution
	Need for closeness to God	Prayer, mosque attendance, engaging in religious conversations, reading the Quran
Personal practices to meet spiritual needs	Worship	Performing prayers, supplication, reciting dhikr, prayer beads
	Relaxing activities	Walking, conversation, reading, contact with family, communicating with other patients
Spiritual care within nursing practice	Relief through nursing interventions	Pain relief, intravenous treatment, medication, vitamin support, comfort measures
	Feeling valued and cared for	Friendliness, responsiveness, attentiveness, asking about needs
Expected spiritual care and limiting factors	Psychosocial supportive approach	Friendliness, communication, emotional support, hope, encouragement
	Barriers to spiritual care	Workload, time constraints, privacy of spirituality, reluctance to share needs

### Perception of spirituality

Participants described spirituality as a deeply personal and multidimensional concept that encompassed both inner emotional states and faith-based beliefs. For some, spirituality was primarily associated with peace and inner comfort, reflecting a sense of psychological stability and freedom from distress. Participant 2 expressed this sentiment clearly:

“For me, spirituality means being filled with peace. Being at peace, having inner comfort, feeling good.”

Similarly, Participant 11 linked spirituality to daily experiences that nourish emotional balance:

“Spirituality is everything that brings peace to a person. My relationship with my children, the times I meet my students, and the books I love all nurture me spiritually.”

For others, spirituality was grounded more explicitly in faith. These participants viewed spirituality as inseparable from religious belief and practice, particularly prayer, worship, and a sense of closeness to God. Participant 7 reflected,

“Spirituality is something that believers possess. It comes from within: for example, through praying.”

Participant 12 stated,

“Spirituality exists in people who have faith in God. Being close to God and fulfilling one’s worship constitute spirituality.”

### Perception of spiritual care

Participants understood spiritual care as both emotional presence and faith-sensitive support within the professional care process. Across narratives, the therapeutic relationship was central. Nurses who demonstrated attentiveness, compassion, and sincere communication were perceived as offering not only physical but also spiritual healing. Participant 3 shared,

“When I think of spiritual care, I think of attention, taking care of the patient, helping them, and providing peace.”

Similarly, Participant 9 emphasized that spiritual care involved offering morale and encouragement:

“Spiritual care is when nurses give morale, offer support, and show care. It brings peace to a person.”

Communication quality also emerged as a defining element. Participants described the power of simple gestures, such as smile, kind tone, or empathetic ear, to foster a sense of spiritual connection. As Participant 5 noted,

“Spiritual care means being cheerful, speaking kindly, and acting without prejudice.”

While spirituality was not always defined in religious terms, some participants explicitly linked spiritual care to faith-based practices. Participant 6 stated,

“When I think of spiritual care, I also think of getting closer to God. Worshiping and praying can be part of it.”

### Spiritual needs during hospitalization

Participants articulated a range of spiritual needs that became more salient during hospitalization. These included the need for psychosocial support, personal hygiene, and closeness to God.

The most frequently expressed need was for psychosocial and emotional support. Many patients longed for family presence, conversation, and words of encouragement to help them maintain hope. Participant 8 shared,

“I needed support, morale, and motivation. It would have been nice if my siblings had visited me, as it would have lifted my spirits.”

Similarly, Participant 10 noted,

“I wanted someone to tell me things like, ‘It will pass, you’ll get through it,’ so I could find hope. Hearing such things makes you feel stronger.”

Maintaining personal hygiene and cleanliness was also viewed as vital for spiritual comfort. Participants emphasized that when they were unable to bathe or perform ablution, their sense of well-being was disturbed. Participant 2 remarked,

“When I first came, I shared a room with another patient, so I couldn’t take a shower or perform ablution. It made me very uncomfortable.”

The need for closeness to God was another key aspect of spiritual life during hospitalization. Several participants expressed sadness over their inability to perform religious practices. Participant 1 explained,

“I need to pray, but I can’t perform ablution and do so properly here, and that makes me sad.”

### Personal practices to meet spiritual needs

Participants described several self-initiated strategies to maintain spiritual well-being during their hospital stay. Many relied on religious worship, such as prayer, supplication, and reciting dhikr, as key coping mechanisms. Participant 6 shared,

“I pray frequently. I cannot always perform the ritual prayer, but I use my prayer beads, reciting ‘Allah’ and sending blessings whenever I remember. It gives me comfort.”

In addition to prayer, participants engaged in relaxing activities such as walking, reading, and talking with others to sustain their emotional balance. Participant 11 explained,

“I make video calls with my children and grandchildren. I have also met new people here. I stay in a single room, but I visit patients in the next room, and we talk. I can say I find peace when I connect and share things with them.”

### Spiritual care within nursing practice

Participants frequently emphasized that spiritual support was not limited to faith-related acts but was also expressed through nursing interventions that alleviated discomfort and fostered dignity. Pain relief, medication administration, and physical comfort measures were described as enhancing spiritual well-being. Participant 2 remarked,

“When I’m in pain, I become restless. Then the nurses give me IV treatment, and that relaxes me, and I feel at peace.”

Participant 12 stated,

“They give me fluids (intravenous medication) and drugs, and these help a lot. Especially at night, they make it easier for me to sleep peacefully.”

Feeling valued and cared for was another recurrent theme. Nurses’ friendliness, reassurance, and attentiveness were perceived as integral to spiritual care. Participant 9 reflected,

“The nurses have always been cheerful with me. They asked how I was and if I needed anything. When I asked questions, they didn’t dismiss me but gave answers.”

### Expected spiritual care and limiting factors

Participants expressed a strong desire for more holistic, compassionate spiritual care that includes psychosocial support, empathy, and acknowledgment of personal worth. Participant 10 suggested,

“Short and pleasant conversations may be beneficial. They (nurses) could support us and tell us that we’ll get better. That would lift our morale and bring spiritual relief.”

Participant 12 explained,

“They could talk to me to help me relax slightly. When you are alone and cannot see your loved ones, you start to feel hopeless. It would be beneficial if they could give me some hope. They are professionals, and hearing good words from someone knowledgeable would really encourage me.”

Despite these expectations, several participants stated that they rarely shared their spiritual needs with nurses, often due to perceptions that such needs were private or secondary. Participant 1 said,

“I didn’t say anything, and I don’t think I needed to. What could they do if I cannot pray? That is something personal; it is not something they can fix.”

Similarly, Participant 11 noted,

“I didn’t specifically mention it; it’s hard to talk about. People often see it as an unnecessary need. Everyone is mostly focused on physical health, not really on how we feel.”

Most participants acknowledged that nurses’ workload and time constraints were the main barriers preventing them from offering more consistent spiritual care. Participant 11 stated,

“Nurses are already working hard to meet our needs... However, as previously mentioned, their workload is excessive. If it were reduced, I believe they could spend more time with us.”

## Discussion

This study explored how patients with prolonged hospitalizations perceive spirituality, spiritual care, and their spiritual needs within a clinical context. The findings highlight the multidimensional nature of spirituality, encompassing both existential and relational dimensions as well as religious beliefs and practices. Participants described spirituality as an inner process of achieving peace, meaning, and emotional balance, while also emphasizing faith-based practices such as prayer, worship, and closeness to God. This pattern suggests that personal and religious dimensions of spirituality are not discrete but interconnected components of a holistic experience. Patients’ accounts revealed a strong overlap between meaning-making processes and religious expressions, suggesting that spirituality was often articulated through faith-based frameworks. This interpretation is consistent with conceptualizations of multidimensional spiritual needs [10].

Consistent with literature, spirituality extends beyond formal religious practices and includes psychological balance, meaning-making, and overall well-being [3,33]. However, the findings also underscore the importance of sociocultural context. Much of the spiritual care literature has been developed in Western settings, where spirituality is often conceptualized as more individualized and less explicitly religious [4]. In contrast, represents an intermediate context as a secular society with a predominantly Muslim cultural background. In this setting, spirituality appears to be expressed through a dynamic interplay of religious and existential dimensions rather than being confined to either domain. This supports previous research indicating that, in culturally Islamic contexts, spiritual experience is often articulated through religious language and practices, even when encompassing broader existential concerns [22,33].

Participants predominantly associated spiritual care with nurses’ attentiveness, communication style, and sensitivity to patients’ religious beliefs. Elements such as therapeutic relationships, active listening, and compassionate interaction highlight that nurse–patient communication constitutes a central component of spiritual care. These findings underscore that spiritual care is not limited to religious facilitation but is also embedded in relational and communicative aspects of nursing practice. At the same time, several participants directly linked spiritual care to worship and closeness to God, indicating that spirituality and spiritual care are often perceived as overlapping constructs. This conceptual overlap has been emphasized in previous research, which positions spirituality and spiritual care as complementary dimensions of holistic nursing and integral to person-centred care [34–36]. Similarly, studies conducted in predominantly Muslim populations demonstrate that spirituality is frequently expressed through worship practices and that these practices are closely integrated into patients’ hospital experiences [37,38]. This suggests that, in such contexts, spiritual care may need to encompass both relational support and facilitation of religious practices to adequately address patients’ needs.

Among the identified spiritual needs, the most prominent theme was the desire for the presence of loved ones and the need for moral and psychosocial support. This finding is consistent with previous research, which has identified psychosocial support as a central source of resilience and coping during hospitalization [39,40]. In addition to psychosocial needs, participants also emphasized the importance of physical aspects of care, particularly personal hygiene. In many accounts, hygiene was described in relation to religious practices such as ablution, suggesting a connection between physical care and the performance of worship. However, as participants’ level of religiosity was not systematically assessed in this study, it is not possible to determine whether these needs were exclusively rooted in religious practice or also reflected broader, non-religious understandings of well-being. This ambiguity indicates that hygiene may function both as a religiously grounded need and as a more general source of comfort and dignity. In this respect, physical care practices can be understood as mediators of spiritual well-being across both religious and non-religious interpretations. This highlights the importance of adopting a context-sensitive and culturally informed approach to holistic nursing care.

Participants also associated their spiritual needs with religious practices such as ablution, prayer, supplication, recitation of dhikr, and attending the mosque. These findings further reinforce the close interconnection between spiritual care and religious practice observed in this study. Previous research indicates that spiritual priorities vary across patient populations and illness contexts. For example, patients with cancer often emphasize intercessory prayer [23,41], while terminally ill individuals may focus on meaning-making and existential questioning [42]. In other contexts, patients prioritize family contact or communication regarding their condition [43]. In addition, the availability of an appropriate environment for prayer has been identified as a significant determinant of spiritual well-being [44]. These findings suggest that spiritual needs are dynamic and shaped by clinical condition, prognostic uncertainty, and sociocultural context. In the present study, the prominence of ritual practices and environmental requirements indicates that spiritual care in this context extends beyond emotional and relational support to include the facilitation of religious practices within the hospital setting.

Personal spiritual practices such as prayer, supplication, and the use of prayer beads (dhikr) reflected patients’ self-initiated strategies to maintain inner peace and cope with uncertainty. This finding aligns with prior research demonstrating that spiritual practices can reduce anxiety and enhance coping capacity in the context of illness and mortality [36,40,45]. However, several participants reported barriers to performing religious rituals, including lack of privacy, shared hospital rooms, limited access to prayer areas, and restricted mobility. These observations highlight that spiritual well-being is shaped not only by individual beliefs and practices but also by the physical and organizational conditions of healthcare settings. Consistent with previous studies, overlapping treatment schedules, immobility, and the presence of medical devices or dressings limited patients’ ability to engage in acts of worship [23,37,46]. Notably, although Islamic jurisprudence provides flexibility in fulfilling religious obligations during illness, some participants perceived this flexibility as permission to suspend their practices entirely [37]. These findings highlight the importance of integrating environmental and institutional considerations into spiritual care. Relatively simple arrangements, including providing prayer rugs, qibla indicators, and access to religious materials, may support patients’ ability to maintain spiritual practices, thereby strengthening their sense of spiritual continuity and improving their overall hospital experience.

Another significant finding was that effective pain management and the provision of physical comfort by nurses were perceived as integral components of spiritual care. This suggests that, from the patients’ perspective, spirituality in nursing extends beyond religious or ritual support to include experiences of comfort, relief, and compassionate presence. Similar observations have been reported in palliative care settings, where practices such as attentive listening, emotional assessment, and therapeutic touch are recognized as expressions of spiritual care [47]. These findings reinforce the understanding that spiritual care is embedded in everyday nursing interactions rather than limited to explicitly religious interventions. These insights highlight the holistic nature of nursing care, in which physical, psychological, and spiritual dimensions are closely interconnected and mutually reinforcing. Addressing physical comfort may therefore contribute not only to symptom relief but also to patients’ spiritual well-being, supporting the integration of spiritual care into routine clinical practice.

Despite the recognized importance of spirituality, most participants reported that they did not share their spiritual needs with nurses. This finding is consistent with previous studies indicating that patients, particularly those with chronic or life-threatening conditions, often hesitate to express spiritual concerns due to time constraints, perceived role boundaries, or fear of burdening healthcare staff [45,48]. In the present study, participants primarily attributed this reluctance to nurses’ heavy workloads. However, the broader literature also identifies additional barriers, including limited competence in spiritual care, low self-efficacy, and the predominance of biomedical priorities in clinical settings [9,49]. These factors collectively contribute to the inconsistent delivery and limited visibility of spiritual care in hospital practice [21]. Failure to recognize and address spiritual needs during prolonged hospitalization may adversely affect not only patients’ psychological well-being but also their overall care experience. In this context, integrating structured spiritual assessments into routine nursing practice, including brief spiritual history-taking at admission and periodic reassessment throughout hospitalization, may facilitate the identification of unmet needs and support their incorporation into holistic care.

### Strengths and limitations

This study provides an in-depth exploration of how patients with prolonged hospitalizations perceive and experience spiritual care within internal medicine settings. The use of a phenomenological design enabled the identification of nuanced, lived experiences that are often not captured through quantitative approaches. The inclusion of participants of different ages and clinical conditions contributed to the diversity of perspectives reflected in the data. In addition, the use of verbatim quotations enhanced the credibility of the findings by grounding interpretations in participants’ own accounts.

Several limitations should be considered. First, the study was conducted in two hospitals within a single urban area of Türkiye, which may limit the transferability of the findings to other cultural or healthcare contexts. Second, the relatively small sample size and the context-specific nature of qualitative research may further constrain generalizability, although the aim was depth rather than breadth of understanding. Third, data were collected solely through self-reported interviews, which may be subject to social desirability or recall bias. Finally, although strategies such as independent coding and consensus discussions were employed to enhance analytic rigor, interpretation remains inherently shaped by researchers’ perspectives. Future research should examine spiritual care by involving nurses, family members, and religious officials across diverse clinical settings. Mixed-methods approaches may further enhance understanding of how spiritual needs are assessed and addressed in hospital care.

## Conclusion

The findings of this study demonstrate that spiritual care cannot be reduced to the facilitation of religious practices alone; rather, it represents a holistic process encompassing nurse–patient communication, psychosocial support, personal hygiene, and sensitivity to patients’ beliefs and values. Prolonged hospitalization disrupts individuals’ daily routines, social support systems, and ability to engage in spiritual practices, thereby intensifying the visibility and significance of spiritual needs.

The barriers identified, such as shared rooms, limited access to prayer spaces, and inadequate hygiene conditions, highlight that the physical and organizational environment constitutes an integral dimension of spiritual care. These findings indicate that spiritual care is a multidimensional and context-dependent phenomenon shaped not only by interpersonal interactions but also by environmental and structural conditions.

In culturally and religiously influenced contexts such as Türkiye, addressing spiritual care requires a comprehensive approach that integrates relational, physical, and environmental aspects into nursing practice. Such an approach has the potential to enhance patients’ spiritual well-being while strengthening the holistic and person-centered nature of care.

### Relevance to clinical practice

The findings of this study indicate that spiritual care can be meaningfully integrated into routine nursing practice through both interpersonal and environmental dimensions of care. Nurses’ everyday interactions, characterized by presence, attentive listening, and respectful communication, provide key opportunities to recognize and respond to patients’ spiritual needs without requiring additional or specialized interventions.

In addition, the findings highlight that spiritual care extends beyond relational support to include physical and environmental conditions that enable patients to sustain their spiritual practices. Simple, context-sensitive arrangements, including ensuring privacy, facilitating access to prayer spaces, supporting personal hygiene, and providing basic religious materials such as prayer rugs or qibla indicators, may significantly enhance patients’ sense of comfort and spiritual well-being.

From a clinical perspective, incorporating a brief spiritual and religious history into initial patient assessments, along with periodic reassessment during prolonged hospital stays, may help identify unmet needs and support their integration into individualized care plans.

At the institutional level, supporting nurses through targeted education, culturally sensitive care training, and manageable workloads is essential to enable meaningful nurse–patient engagement. Integrating spiritual care into routine assessment and care processes may contribute to more holistic, patient-centered care, particularly for individuals experiencing prolonged hospitalization.

## Supporting information

S1 Appendix COREQ checklist. (PDF)

S2 Appendix Semi-structured interview protocol. (PDF)

## References

[1] Reinert KG, Koenig HG. Re-examining definitions of spirituality in nursing research. J Adv Nurs. 2013;69(12):2622–34. doi: 10.1111/jan.12152 23600849 PMC4232181

[2] Heidari A, Afzoon Z, Heidari M. The correlation between spiritual care competence and spiritual health among Iranian nurses. BMC Nurs. 2022;21(1):277. doi: 10.1186/s12912-022-01056-0 36224620 PMC9555262

[3] Puchalski CM, Vitillo R, Hull SK, Reller N. Improving the spiritual dimension of whole person care: reaching national and international consensus. J Palliat Med. 2014;17(6):642–56. doi: 10.1089/jpm.2014.9427 24842136 PMC4038982

[4] LeDoux J, Mann C, Demoratz M, Young J. Addressing Spiritual and Religious Influences in Care Delivery. Prof Case Manag. 2019;24(3):142–7. doi: 10.1097/NCM.0000000000000346 30946252

[5] Sabet P, Karimi S, Dehghan A, Bijani M. Effect of spirituality-based palliative care on pain, nausea, vomiting, and quality of life in women with colon cancer. J Relig Health. 2023;62(3):1985–97. doi: 10.1007/s10943-023-01742-6 36809520

[6] Safdar MR, Akram M, Ahmad A, Ayaz AA. Role of religion and spirituality in coping with COVID-19 among people of low socioeconomic status in Pakistan. J Relig Health. 2023;62(4):2916–32. doi: 10.1007/s10943-023-01781-z 36877419 PMC9987379

[7] Relawati A, Rochmawati E, Primanda Y, Kamil AR, Arianti, Haris F, et al. Spiritual Interventions to Improve Quality of Life and Spiritual Well-Being: A Systematic Review and Meta-analysis. J Relig Health. 2025;64(5):3346–64. doi: 10.1007/s10943-025-02333-3 40517192

[8] Sun M, Tian X, Peng Y, Wang Z, Lu Y, Xiao W. Effects of meaning therapy on spirituality, psychological health, and quality of life in patients with cancer: A systematic review and meta-analysis of randomized controlled trials. Asia Pac J Oncol Nurs. 2024;11(4):100388. doi: 10.1016/j.apjon.2024.100388 38586470 PMC10997828

[9] Alrukban M, Alrabiah A, Alomri F, Alghuligah A, Alderaywsh A, Alomar A, et al. The perception of spirituality and its assessment among those with different health statuses in Saudi Arabia. Healthcare (Basel). 2023;11(14):2034. doi: 10.3390/healthcare11142034 37510475 PMC10379964

[10] Kellehear A. Spirituality and palliative care: a model of needs. Palliat Med. 2000;14(2):149–55. doi: 10.1191/026921600674786394 10829149

[11] Al-Ghabeesh SH, Alshraifeen AA, Saifan AR, Bashayreh IH, Alnuaimi KM, Masalha HA. Spirituality in the Lives of Patients with End-Stage Renal Disease: A Systematic Review. J Relig Health. 2018;57(6):2461–77. doi: 10.1007/s10943-018-0622-2 29671169

[12] Quinn M, Fowler KE, Harrod M, Ehrlinger R, Engle JM, Houchens N, et al. Exploring sacred moments in hospitalized patients: an exploratory qualitative study. J Gen Intern Med. 2023;38(9):2038–44. doi: 10.1007/s11606-022-07999-z 36650333 PMC9845021

[13] Mathew L, Kunnath B. Unmet Spiritual Needs: A Study among Patients with Chronic Illness. Indian J Palliat Care. 2024;30(4):342–6. doi: 10.25259/IJPC_39_2024 39650586 PMC11618673

[14] Arshinoff R, Roldan C, Balboni T. Spirituality and spiritual distress in neurologic illness. Handb Clin Neurol. 2023;191:221–34. doi: 10.1016/B978-0-12-824535-4.00004-5 36599510

[15] Asadzandi M. An Islamic Religious Spiritual Health Training Model for Patients. J Relig Health. 2020;59(1):173–87. doi: 10.1007/s10943-018-0709-9 30311051

[16] Taylor EJ, Mamier I, Ricci-Allegra P, Foith J. Self-reported frequency of nurse-provided spiritual care. Appl Nurs Res. 2017;35:30–5. doi: 10.1016/j.apnr.2017.02.019 28532723

[17] Blasdell ND. The evolution of spirituality in the nursing literature. Int J Caring Sci. 2015;8(3):756–64.

[18] International Council of Nurses. The ICN code of ethics for nurses. Geneva: ICN. 2021.

[19] Southard ME. Spirituality: The Missing Link for Holistic Health Care. J Holist Nurs. 2020;38(1):4–7. doi: 10.1177/0898010119880361 32253989

[20] Voetmann SS, Hvidt NC, Viftrup DT. Verbalizing spiritual needs in palliative care. BMC Palliat Care. 2022;21(1):3. doi: 10.1186/s12904-021-00886-0 34980085 PMC8725243

[21] Wang W, Yang J, Bai D, Lu X, Gong X, Cai M, et al. Nurses’ perceptions and competencies about spirituality and spiritual care: A systematic review and meta-analysis. Nurse Educ Today. 2024;132:106006. doi: 10.1016/j.nedt.2023.106006 37922766

[22] Mushtaq R, Hasan SS, Khadim R. Lived experiences of adults about religious orientation and spirituality. J Prof Appl Psychol. 2023;4(2):266–80. doi: 10.52053/jpap.v4i2.187

[23] ACAR G, SAĞKAL MİDİLLİ T. Examination of Spiritual Care Needs of Oncology Patients and Spiritual Care Competencies of Oncology Nurses. İzmir Katip Çelebi Üniversitesi Sağlık Bilimleri Fakültesi Dergisi. 2023;8(3):917–23. doi: 10.61399/ikcusbfd.1134673

[24] Kurtgöz A, Genç M. Spiritual care perspectives of elderly individuals with parkinson’s disease and formal caregivers: a qualitative study in turkish nursing homes. J Relig Health. 2024;63(3):2106–24. doi: 10.1007/s10943-023-01963-9 38042960

[25] Creswell JW. Qualitative inquiry and research design: choosing among five approaches. 3rd ed. Thousand Oaks (CA): Sage Publications. 2013.

[26] Tong A, Sainsbury P, Craig J. Consolidated criteria for reporting qualitative research (COREQ): a 32-item checklist for interviews and focus groups. Int J Qual Health Care. 2007;19(6):349–57. doi: 10.1093/intqhc/mzm042 17872937

[27] Woodward T, Josephson C, Ross L, Hill J, Hosking B, Naumann F, et al. A retrospective study of the incidence and characteristics of long-stay adult inpatients with hospital-acquired malnutrition across five Australian public hospitals. Eur J Clin Nutr. 2020;74(12):1668–76. doi: 10.1038/s41430-020-0648-x 32393753

[28] Creswell JW, Plano Clark VL. Designing and conducting mixed methods research. 3rd ed. Los Angeles (CA): Sage Publications. 2018.

[29] Cohen DJ, Crabtree BF. Evaluative criteria for qualitative research in health care: controversies and recommendations. Ann Fam Med. 2008;6(4):331–9. doi: 10.1370/afm.818 18626033 PMC2478498

[30] Patton MQ. Qualitative research & evaluation methods: Integrating theory and practice. 4th ed. Sage Publications. 2015.

[31] Nowell LS, Norris JM, White DE, Moules NJ. Thematic analysis: striving to meet the trustworthiness criteria. Int J Qual Methods. 2017;16(1):1–13. doi: 10.1177/1609406917733847

[32] Ortlipp M. Keeping and using reflective journals in the qualitative research process. Qual Rep. 2008;13(4):695–705. doi: 10.46743/2160-3715/2008.1579

[33] Koenig HG. Religion and mental health: research and clinical applications. London: Elsevier Academic Press. 2018.

[34] Devi MK, Fong KCK. Spiritual Experiences of Women with Breast Cancer in Singapore: a Qualitative Study. Asia Pac J Oncol Nurs. 2019;6(2):145–50. doi: 10.4103/apjon.apjon_77_18 30931358 PMC6371664

[35] Kang K-A, Kim S-J. Comparison of perceptions of spiritual care among patients with life-threatening cancer, primary family caregivers, and hospice/palliative care nurses in South Korea. J Hosp Palliat Nurs. 2020;22(6):532–51. doi: 10.1097/NJH.0000000000000697 33044420

[36] Sajadi M, Niazi N, Khosravi S, Yaghobi A, Rezaei M, Koenig HG. Effect of spiritual counseling on spiritual well-being in Iranian women with cancer: A randomized clinical trial. Complement Ther Clin Pract. 2018;30:79–84. doi: 10.1016/j.ctcp.2017.12.011 29389484

[37] Abdul Halim RZ, Saidi S, Mohd Yusof N, Che Ahmad A, Hassan NH, Suryanne S S. Performing obligatory prayer while hospitalised: perspectives of muslim patients in a teaching hospital in Malaysia. IJICI. 2024;9(2):1306–19. doi: 10.53840/alirsyad.v9i2.458

[38] Irajpour A, Moghimian M, Arzani H. Spiritual aspects of care for chronic Muslim patients: A qualitative study. J Educ Health Promot. 2018;7:118. doi: 10.4103/jehp.jehp_199_17 30271803 PMC6149117

[39] Ali G, Snowden M, Wattis J, Rogers M. Spirituality in nursing education: knowledge and practice gaps. Int J Multidiscip Comp Stud. 2018;5(1–3):27–49.

[40] Hosseini FA, Momennasab M, Yektatalab S, Zareiyan A. Spiritual needs of surgical patients in Iranian hospital settings. BMC Res Notes. 2025;18(1):155. doi: 10.1186/s13104-025-07228-w 40205456 PMC11983834

[41] Miller M, Galchutt P, Meyers M, Rosa WE. Understanding meanings and lived experiences of spirituality among adults with cancer. J Psychosoc Oncol. 2026;44(2):149–73. doi: 10.1080/07347332.2025.2541643 40765158 PMC12360301

[42] Clyne B, O’Neill SM, Nuzum D, O’Neill M, Larkin J, Ryan M, et al. Patients’ spirituality perspectives at the end of life: a qualitative evidence synthesis. BMJ Support Palliat Care. 2022;12(e4):e550–61. doi: 10.1136/bmjspcare-2019-002016 31771958

[43] Yazgan E, Demir A. Factors affecting the tendency of cancer patients for religion and spirituality: a questionnaire-based study. J Relig Health. 2019;58(3):891–907. doi: 10.1007/s10943-017-0468-z 28849378

[44] Yousefi H, Abedi HA. Spiritual care in hospitalized patients. Iran J Nurs Midwifery Res. 2011;16(1):125–32. 22039390 PMC3203292

[45] Dewi I, Sastro R, Alamsyah S. Relationship between nurses’ characteristics and implementation of Islamic spiritual nursing care in adult inpatient wards of Bandung district hospitals. J Smart Keperawatan. 2020;7(2):125. doi: 10.34310/jskp.v7i2.346

[46] Sastra L, Büssing A, Chen C-H, Yen M, Lin EC-L. Spiritual Needs and Influencing Factors of Indonesian Muslims With Cancer During Hospitalization. J Transcult Nurs. 2021;32(3):212–20. doi: 10.1177/1043659620908926 32167014

[47] Rocha RCNP, Pereira ER, Silva RMCRA, Medeiros AYBBV de, Refrande SM, Refrande NA. Spiritual needs experienced by the patient’s family caregiver under Oncology palliative care. Rev Bras Enferm. 2018;71(suppl 6):2635–42. doi: 10.1590/0034-7167-2017-0873 30540038

[48] Candan Z, Uğur Ö. Spiritual well-being of cancer patients in Turkey and associated factors. Acibadem Univ Health Sci J. 2023;14(2):147–54. doi: 10.31067/acusaglik.1217464

[49] Sari NK, Asriani AD. Islamic nursing care implementation: patients’ perception. Bali Med J. 2023;12(1):556–9. doi: 10.15562/bmj.v12i1.3780

