# Supplementary material for: Patients’ perception and needs of spiritual care: A qualitative study in the context of prolonged hospitalizations
Source: PLoS One. 2026 Apr 24;21(4):e0347552. doi: 10.1371/journal.pone.0347552 (PMC13108811; doi:10.1371/journal.pone.0347552)
Supplement: S1 Appendix — (PDF) [file pone.0347552.s001.pdf]

COREQ checklist

Item No	Guide Questions/Description	Reported on Page
**Domain 1: Research team and reflexivity**		
**Personal Characteristics**		
1. Interviewer/ facilitator	Which author/s conducted the interview or focus group?	Page 6-7, Data Collection
2. Credentials	What were the researcher's credentials? E.g., PhD, MD	Page 8, Data Analysis
3. Occupation	What was their occupation at the time of the study?	Page 8, Data Analysis
4. Gender	Was the researcher male or female?	Female, Page 6
5. Experience and training	What experience or training did the researcher have?	Page 8, Data Analysis
**Relationship with participants**		
6. Relationship established	Was a relationship established prior to study commencement?	Page 6-7, Data Collection
7. Participant knowledge of the interviewer	What did the participants know about the researcher? e.g. personal goals, reasons for doing the research?	Page 9-10, Ethical Considerations
8. Interviewer characteristics	What characteristics were reported about the interviewer/facilitator? e.g. Bias, assumptions, reasons and interests in the research topic	Page 8, Data Analysis
**Domain 2: study design**		
**Theoretical framework**		
9. Methodological orientation and Theory	What methodological orientation was stated to underpin the study? e.g. grounded theory, discourse analysis, ethnography, phenomenology, content analysis	Page 5, Study Design
**Participant selection**		
10. Sampling	How were participants selected? e.g., purposive, convenience, consecutive, snowball	Page 5-6, Sample

Item No	Guide Questions/Description	Reported on Page
11. Method of approach	How were participants approached? e.g., face-to-face, telephone, mail, email	Page 7, Data Collection
12. Sample size	How many participants were in the study?	Page 6, Study Setting and Sample
13. Non-participation Setting	How many people refused to participate or dropped out? Reasons?	Page 7, Data Collection
14. Setting of data collection	Where was the data collected? e.g., home, clinic, workplace	Page 7, Data Collection
15. Presence of nonparticipants	Was anyone else present besides the participants and researchers?	Page 7, Data Collection
16. Description of sample	What are the important characteristics of the sample? e.g. demographic data, date	Page 10-11, Results<br>Table 1
Data collection		
17. Interview guide	Were questions, prompts, and guides provided by the authors? Was it pilot tested?	Interview Guide / Supplement
18. Repeat interviews	Were repeat interviews carried out? If yes, how many?	No
19. Audio/visual recording	Did the research use audio or visual recording to collect the data?	Page 7, Data Collection
20. Field notes	Were field notes made during and/or after the interview or focus group?	Page 7, Data Collection
21. Duration	What was the duration of the interviews or focus group?	Page 7, Data Collection
22. Data saturation	Was data saturation discussed?	Page 6, Study Setting and Sample
23. Transcripts returned	Were transcripts returned to participants for comment and/or correction?	No
Domain 3: analysis and findings		
Data analysis		
24. Number of data coders	How many data coders coded the data?	Page 8, Data Analysis
25. Description of the coding tree	Did the authors provide a description of the coding tree?	No
26. Derivation of themes	Were themes identified in advance or derived from the data?	Page 8, Data Analysis
27. Software	What software, if applicable, was used to manage the data?	No
28. Participant checking	Did participants provide feedback on the findings?	No

Item No	Guide Questions/Description	Reported on Page
**Reporting**		
29. Quotations presented	Were participant quotations presented to illustrate the themes/findings? Was each quotation identified? e.g., participant number	Page 12-17, Results
30. Data and findings consistent	Was there consistency between the data presented and the findings?	Table 2, Results
31. Clarity of major themes	Were major themes clearly presented in the findings?	Table 2, Results Page 12-17
32. Clarity of minor themes	Is there a description of diverse cases or a discussion of minor themes?	Page 17-22, Discussion
