# Supplementary material for: Patients’ perception and needs of spiritual care: A qualitative study in the context of prolonged hospitalizations
Source: PLoS One. 2026 Apr 24;21(4):e0347552. doi: 10.1371/journal.pone.0347552 (PMC13108811; doi:10.1371/journal.pone.0347552)
Supplement: S2 Appendix — (PDF) [file pone.0347552.s002.pdf]

Semi-Structured Interview Protocol

Participant number:
Date of interview:
Location:
Start Time:
End Time:

Pre-interview procedures

- Explained the purpose of the study.
- Obtained written consent from the participant.
- Ensured that the participant felt comfortable and ready to begin the interview.
- Reminded the participant that there were no right or wrong answers.
- Emphasized that the participant's experiences and opinions were valued and respected.

Socio-demographic and clinical information

- Gender and age
- Marital status and number of children (if any)
- Educational background
- Occupation and employment status
- Diagnosis and duration of illness
- Number of previous hospitalizations
- Duration of current hospital stay

Understanding spirituality and spiritual care

- What does spirituality mean to you? How would you describe it in your own words?
- What does spiritual care mean to you?
- Before your hospitalization, what kinds of activities or practices helped you find spiritual comfort or peace at home or in your daily life?
- During your current hospital stay, what have you needed most in terms of spiritual support?
- Have there been any specific practices or experiences that have helped you feel spiritually at ease during your hospital stay? If yes, could you describe them?

Addressing spiritual care needs

- During your hospital stay, have you been able to share your spiritual or religious needs with healthcare providers or nurses?
- Have you received any form of spiritual care from nurses or physicians beyond routine clinical care?
- What kind of things have nurses or doctors done to support your spiritual well-being?
- Considering your overall hospital experience, what do you think nurses could do to better meet patients' (or your own) spiritual care needs?
- In your opinion, what conditions or environmental factors in the hospital influence the delivery of spiritual care?

Additional questions

Interview notes
